# Radiation and Local Anti-CD40 Generate an Effective *in situ* Vaccine in Preclinical Models of Pancreatic Cancer

**DOI:** 10.3389/fimmu.2018.02030

**Published:** 2018-09-07

**Authors:** Sayeda Yasmin-Karim, Patrick T. Bruck, Michele Moreau, Sijumon Kunjachan, Gui Zhen Chen, Rajiv Kumar, Stephanie Grabow, Stephanie K. Dougan, Wilfred Ngwa

**Affiliations:** ^1^Department of Radiation Oncology, Brigham and Women's Hospital, Boston, MA, United States; ^2^Department of Radiation Oncology, Harvard Medical School, Boston, MA, United States; ^3^Department of Cancer Immunology and Virology, Dana-Farber Cancer Institute, Boston, MA, United States; ^4^Department of Biology, University of Massachusetts, Lowell, MA, United States; ^5^Electronic Materials Research Institute, Northeastern University, Boston, MA, United States; ^6^Department of Microbiology and Immunobiology, Harvard Medical School, Boston, MA, United States

**Keywords:** radiotherapy, abscopal effect, immunotherapy, pancreatic cancer, CD40, vitiligo

## Abstract

Radiation therapy induces immunogenic cell death, which can theoretically stimulate T cell priming and induction of tumor-specific memory T cell responses, serving as an *in situ* vaccine. In practice, this abscopal effect is rarely observed. We use two mouse models of pancreatic cancer to show that a single dose of stereotactic body radiation therapy (SBRT) synergizes with intratumoral injection of agonistic anti-CD40, resulting in regression of non-treated contralateral tumors and formation of long-term immunologic memory. Long-term survival was not observed when mice received multiple fractions of SBRT, or when TGFβ blockade was combined with SBRT. SBRT and anti-CD40 was so effective at augmenting T cell priming, that memory CD8 T cell responses to both tumor and self-antigens were induced, resulting in vitiligo in long-term survivors.

## Introduction

Successful generation of an anti-tumor CD8 T cell response involves multiple steps. First, local dendritic cells, laden with antigens from dying tumor cells, become activated and migrate to the draining lymph node (1). There, activated dendritic cells interact with naïve T cells which become primed, proliferate, and acquire effector capabilities. These activated effector T cells then traffic to the tumor, and ideally are able to kill tumor cells via direct cytolysis or production of interferon (IFN)γ. Immunosuppressive myeloid cells in the tumor microenvironment, as well as nutrient starvation and expression of inhibitory ligands such as PD-L1, may prevent CD8 T cell-mediated killing even when CD8 T cell priming has occurred. The fact that immune checkpoint blockade has single-agent efficacy in some cancer patients indicates that CD8 T cell priming successfully occurs in a significant fraction of humans with cancer (2, 3).

However, anti-tumor CD8 T cells are not found in all patients, and therapeutic cancer vaccines have been developed to induce T cell priming *de novo* (4–6). Systemic vaccines require knowledge of the antigens of interest, or at a minimum, cumbersome preparation of tumor cell lysates. Perhaps the simplest and most effective vaccination strategies involve direct delivery of immune stimulatory agents to the tumor microenvironment (7). These so-called “*in situ*” vaccines operate under the idea that induction of tumor cell death releases tumor antigens, which are phagocytosed and presented by local dendritic cells that become activated and prime naïve T cells in the draining lymph node (1). Successful *in situ* vaccines require both a means of tumor cell death and a source of adjuvant to activate local dendritic cells. Oncolytic viruses serve both functions, and local injection of TVEC is approved for metastatic melanoma patients (8, 9). Local delivery of adjuvants such as STING agonists or TLR ligands have been proposed, although these agents do not induce cell death on their own, and may be more efficacious when combined with radiation or with certain chemotherapies or targeted therapies (7, 10–13).

Radiation has long been used to treat cancer patients, usually for local control or palliation (14). In rare cases, regression of lesions outside the field of radiation have been observed (14, 15). This so-called abscopal effect is due to induction of adaptive immunity and recognition of tumor antigens at distant sites by effector CD8 T cells. Although many agents that induce cell death may be predicted to synergize with immunotherapy, radiation may be particularly good at inducing T cell priming. Radiation has pleiotropic effects on the tumor microenvironment, including induction of MHC expression on tumor cells and upregulation of costimulatory ligands on dendritic cells (16, 17). Indeed, several studies have shown that radiation broadens the oligoclonality of the T cell response, presumably by inducing T cell responses against a wider array of tumor antigens (18, 19). At the same time, radiation induces production of myeloid cell attracting chemokines such as CCL2 that can establish an immunosuppressive microenvironment (20). Combination of radiation and immune stimulating adjuvants is therefore critical.

CD40 is a TNF family member expressed on dendritic cells, macrophages and B cells. When engaged by CD40L or by an agonistic antibody, CD40 signaling leads to NF-κB upregulation and expression of costimulatory ligands, production of IL-12 and other cytokines, enhanced antigen presentation, and in the case of dendritic cells, upregulation of CCR7, and trafficking to the draining lymph node. Agonistic antibodies to CD40 have been successful in generating limited responses in both mice and humans with pancreatic tumors, in some cases via enhanced T cell priming, and in other cases through activation of myeloid cells (21–24). In mouse models of pancreatic ductal adenocarcinoma, SBRT was shown to transiently deplete CD8 T cells, increase MHC class I expression on tumor cells and be synergistic with checkpoint blockade (18, 25). SBRT combined with systemically delivered anti-CD40, anti-PD1, and anti-CTLA4 led to durable remissions of the majority of subcutaneous tumors, in a manner that was dependent on endogenously primed T cells and IFNγ (25), although the dual combination of SBRT and anti-CD40 was not evaluated. In pancreatic neuroendocrine tumors, radiation, and agonistic anti-CD40 together were insufficient to induce T cell priming, although these two agents served as preconditioning regimens for successful adoptive T cell therapy (26).

Pancreatic tumors are notoriously refractory to therapy, including immunotherapy (27). Adjuvants that stimulate dendritic cell activation and T cell priming in other cancer types may have tumor promoting effects in pancreatic cancer. Pancreatic tumor cells constitutively express TLR7, secrete myeloid cell recruitment and maturation factors such as GM-CSF, and have chronic STING pathway activation due to chromothryptic events and the formation of micronuclei (28–32). TGFβ blockade is effective at inducing CD8 T cell influx (33, 34), and synergizes with radiation in other tumor types (35, 36); however whether blockade of TGFβ signaling in pancreatic tumors would synergize with radiation is unclear given that pancreatic cancer cells rely on TGFβ signaling to maintain radiosensitivity (37).

Here we defined the effects of radiotherapy on anti-tumor immunity in two mouse models of pancreatic cancer. A single moderate dose of stereotactic body radiotherapy (SBRT), along with intratumoral injection of agonistic anti-CD40 induced complete regressions in both treated and non-treated lesions. Tumor regression was associated with decreased myeloid populations and increased percentages of CD8 T cells. Cured mice were refractory to rechallenge, indicating successful generation of immunologic memory. CD8 T cell priming was robustly induced, with mice generating not only anti-tumor T cells, but also auto-reactive T cells capable of inducing vitiligo.

## Results

### Single but not multiple dose SBRT combined with intratumoral anti-CD40 leads to regression of contralateral Panc02 pancreatic tumors

**Figure 1:**
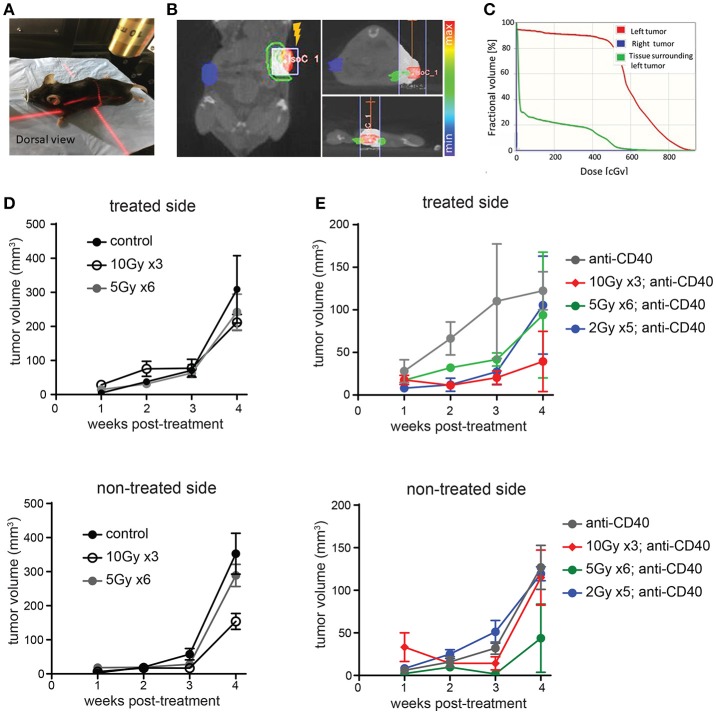
Multiple fractions of image guided SBRT delivers radiation precisely to pancreatic tumors, but fails to achieve an abscopal effect. **(A)** A Small Animal Radiation Research Platform (SARRP) was used for RT. Image guided RT was given to the left tumor only. **(B,C)** Dosimetry showing the CT view of a mouse during image guided RT and dosimetry showing RT distribution in treated and contralateral tumors, as well as surrounding normal tissue. **(D)** C57BL/6 mice were inoculated with Panc02 tumors on each flank. Once tumors were palpable, mice were treated on one flank with no SBRT, 10Gy on three consecutive days, or 5Gy on 6 consecutive days. Tumor growth on each side was measured. *n* = 5 mice/group. **(E)** Mice were treated as in **(D)**, except that anti-CD40 was administered (10 μg, intratumoral) with the first and last dose of SBRT, or two injections 5 days apart in mice receiving no SBRT. *n* = 5 mice/group. Error bars are SD.

We used image guidance to deliver precise doses of SBRT to defined areas in mice using a small animal radiation research platform (SARRP) (Figures 1A–C). Mice bearing subcutaneous Panc02 tumors on each flank were treated unilaterally with 5 × 2 Gy, 6 × 5Gy, or 3 × 10 Gy. Pancreatic tumors are relatively resistant to radiotherapy, and both irradiated and non-irradiated lesions grew progressively (Figure 1D and Supplemental Figure 1). Addition of intratumoral anti-CD40 administered concurrently with the first and last fractions of SBRT improved local control of treated tumors at the 10Gy dose, but did not induce regression of contralateral tumors (Figure 1E).

**Figure 2:**
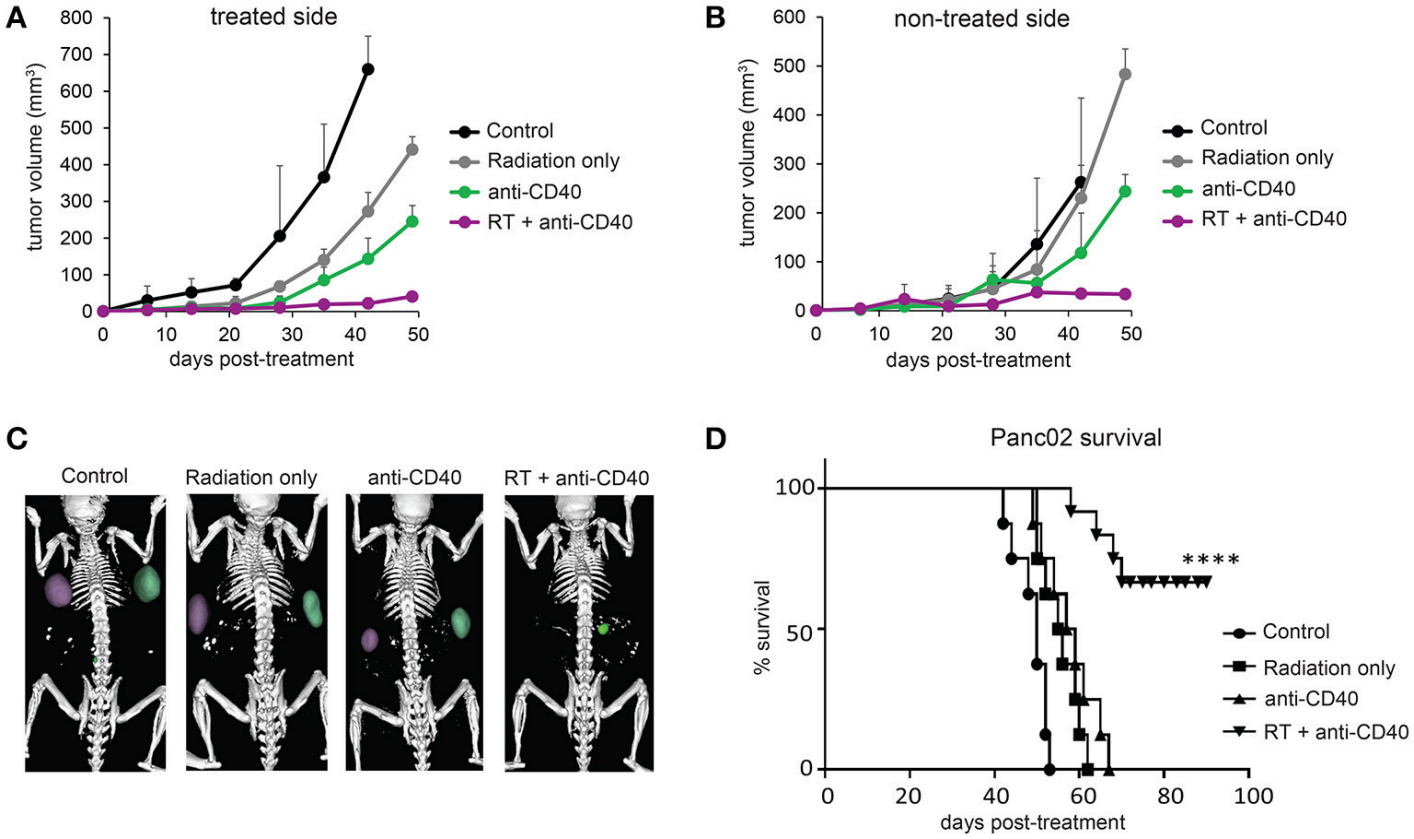
Single dose 5Gy SBRT combined with anti-CD40 induces regression of contralateral Panc02 tumors. C57BL/6 mice were inoculated with Panc02 tumors on each flank. Once tumors reached palpable size, the right flank was treated with RT and/or a single dose of anti-CD40 (20 μg) as indicated. **(A)** Volumes of treated tumors over time, measured by CT. **(B)** Volumes of contralateral tumors over time, measured by CT. **(C)** Representative CT imaging of mice at 3 weeks post-treatment. **(D)** Overall survival. *n* = 8/group. ^****^p < 0.0001.

Radiation damages not only tumor cells, but also immune cells that may be present. In the case of radioresistant pancreatic tumors, additional fractions of radiation have little impact on the overall tumor burden. Previous reports of fractionated radiation combined with immunotherapy used checkpoint blockade immunotherapies, which act on T cells that infiltrate tumors a week or more after treatment and are thus temporally protected from the damaging effects of radiation. We hypothesized that multiple fractions of SBRT delivered over several days may be detrimental to local dendritic cells which are required for crosspresentation of tumor antigens to naïve CD8 T cells and are likely the cellular targets of anti-CD40 (38). To address this issue, single dose SBRT of 5Gy with or without intratumoral anti-CD40 was administered to mice bearing Panc02 tumors. Therapy was initiated 2 weeks post-implantation, at a time when all tumors were palpable (~25 mm^3^). SBRT and anti-CD40 administration alone each provided some local control of the treated tumor, but complete regressions of the contralateral tumors were only observed in mice receiving combination SBRT and anti-CD40 (Figures 2A–C). Mice were followed long-term, and overall survival was 80% in the combination group vs. zero in control or single agent treated mice (Figure 2D). We therefore used single dose SBRT in all subsequent experiments.

### Combination therapy induces CD8 T cell infiltration in Panc02 tumors

**Figure 3:**
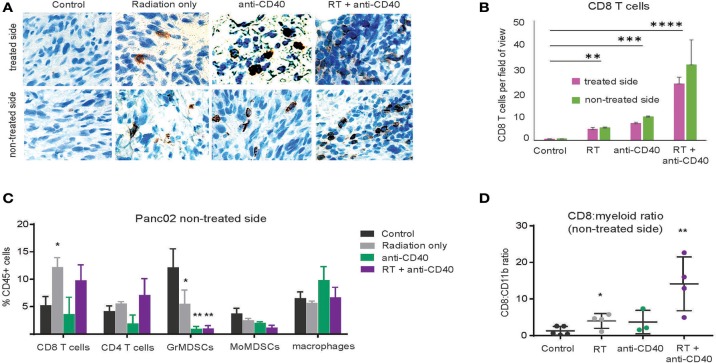
Combination RT and anti-CD40 leads to increased intratumoral CD8 T cells. **(A)** Tumors from mice treated with 5Gy SBRT and/or a single dose of anti-CD40 (20 μg) were harvested at 2 weeks post-treatment and analyzed by immunohistochemistry for CD8. **(B)** Quantification of a. *n* = 5 mice/group. **(C)** Tumors from mice treated as in Figure 2 were harvested at 2 weeks post-treatment, digested and analyzed by flow cytometry. GrMDSCs: CD11b^+^Gr1^high^; MoMDSCs: CD11b^+^Ly6C^+^; macrophages: CD11b^+^,Gr1^−^. **(D)** Ratio of CD8 T cells to total CD11b^+^ myeloid cells. *n* = 5 mice/group. Error bars are SD. ^*^p < 0.05, ^**^p < 0.01, ^***^p < 0.001, ^****^p < 0.0001.

Although CD8 T cells can mediate tumor rejection, they are largely excluded from pancreatic tumors at baseline due, at least in part, to immunosuppressive macrophages (39). Two weeks following therapy, we examined CD8 T cell infiltrates in treated and contralateral Panc02 tumors by histology (Figures 3A,B) and by flow cytometry (Figures 3C,D). Consistent with previous reports, CD8 T cells were infrequent in the interior of control tumors (39). Radiation led to an increase in intratumoral CD8 T cells in both RT and combination treated mice at 3 weeks post therapy. Flow cytometry revealed a decrease in granulocytic (Gr1^high^, CD11b^+^) and monocytic (Ly6C^+^CD11b^+^) myeloid suppressor cells in response to anti-CD40, resulting in an increased CD8 to CD11b ratio that was most striking in the combination treated group. Increased CD8 T cell infiltration was observed in both treated and contralateral tumors, suggesting that CD8 T cells primed against tumor antigens from one tumor were capable of accumulating in non-treated tumors expressing similar antigens.

### Combination SBRT with intratumoral anti-CD40, but not TGFβ blockade, leads to regression of contralateral KPC pancreatic tumors, and formation of immunologic memory

**Figure 4:**
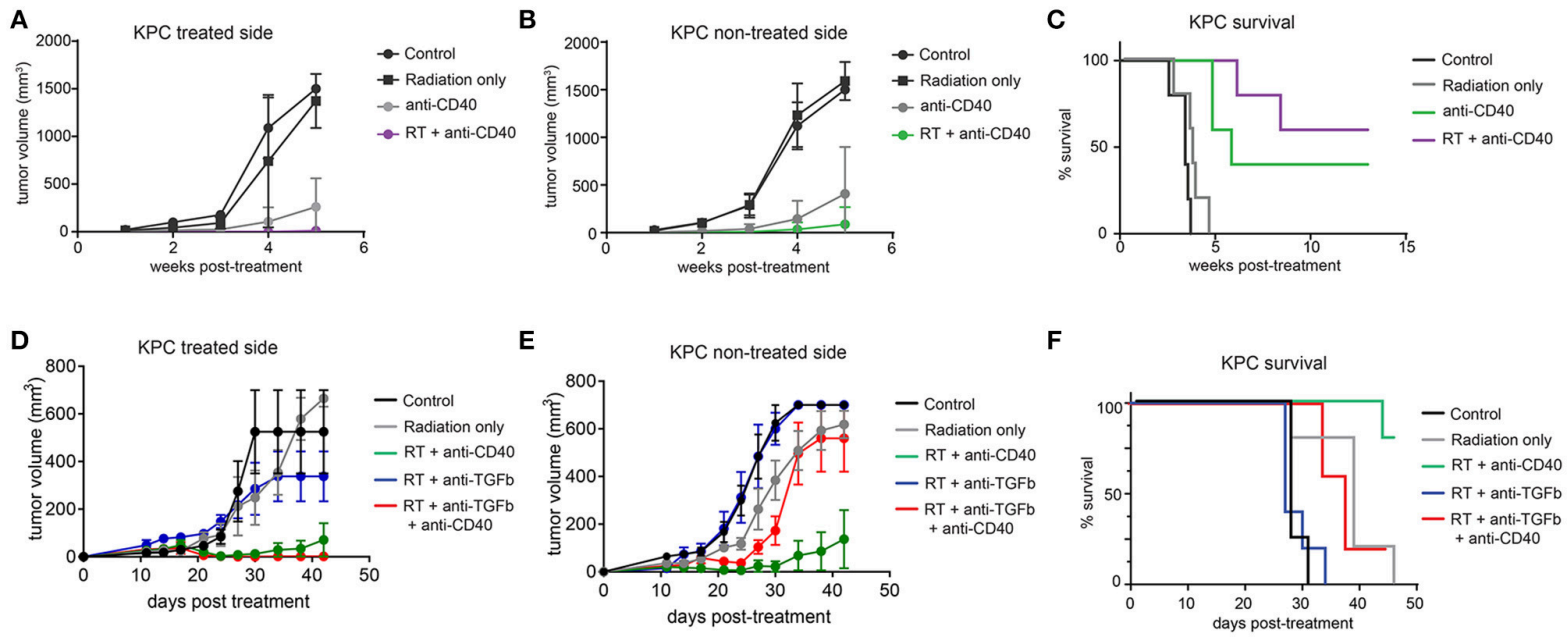
Combination 10Gy RT and anti-CD40 induces regression of contralateral KPC tumors, but TGFβ blockade counteracts the abscopal effect of anti-CD40. C57BL/6 mice were inoculated on each flank with 150,000 KPC cells. Once tumors reached palpable size (11–14 days post-implantation), mice were treated with 10Gy SBRT, anti-CD40 (20 μg once, intratumoral), both RT and anti-CD40, or PBS control. **(A)** Volume of treated side tumors over time. **(B)** Volume of contralateral tumors over time. **(C)** Overall survival. **(D–F)** C57BL/6 mice were treated as in **(A–C)**, except anti-TGFβ (200μg intraperitoneal every 3 days starting at the time of SBRT) was included where indicated. *n* = 5/group. Error bars are SEM.

The Panc02 cell line is notable for a high mutational burden and increased susceptibility to CD8 T cell responses. To better model pancreatic tumors with lower endogenous CD8 T cell responses, we used a cell line derived from the *LSL-Kras;p53*+*/floxed,Pdx-cre* mouse (KPC). These tumors grow similarly in both immunodeficient and immune competent mice, and are resistant to T cell augmenting therapies (40). We tested a similar regimen of single dose SBRT (10Gy) with or without intratumoral anti-CD40 in mice bearing palpable KPC pancreatic tumors on each flank, and again observed significant regression of non-treated tumors and increased overall survival in combination treated mice (Figures 4A–C).

TGFβ has been reported to synergize with radiation therapy in mouse models of breast cancer (36, 41). Furthermore, TGFβ has been shown to restrict CD8 T cells to the periphery of tumors (33), and TGFβ production in pancreatic cancer leads to increased fibroblast activation and stromal deposition, both of which are likely tumor promoting (27). We therefore administered systemic blocking antibodies to TGFβ in combination with SBRT with or without anti-CD40. Contrary to expectations, TGFβ blockade had no effect when combined with SBRT, and triple combination of SBRT, anti-CD40, and TGFβ blockade resulted in regression of the treated tumor, but complete loss of efficacy at the contralateral lesion (Figures 4D–F).

**Figure 5:**
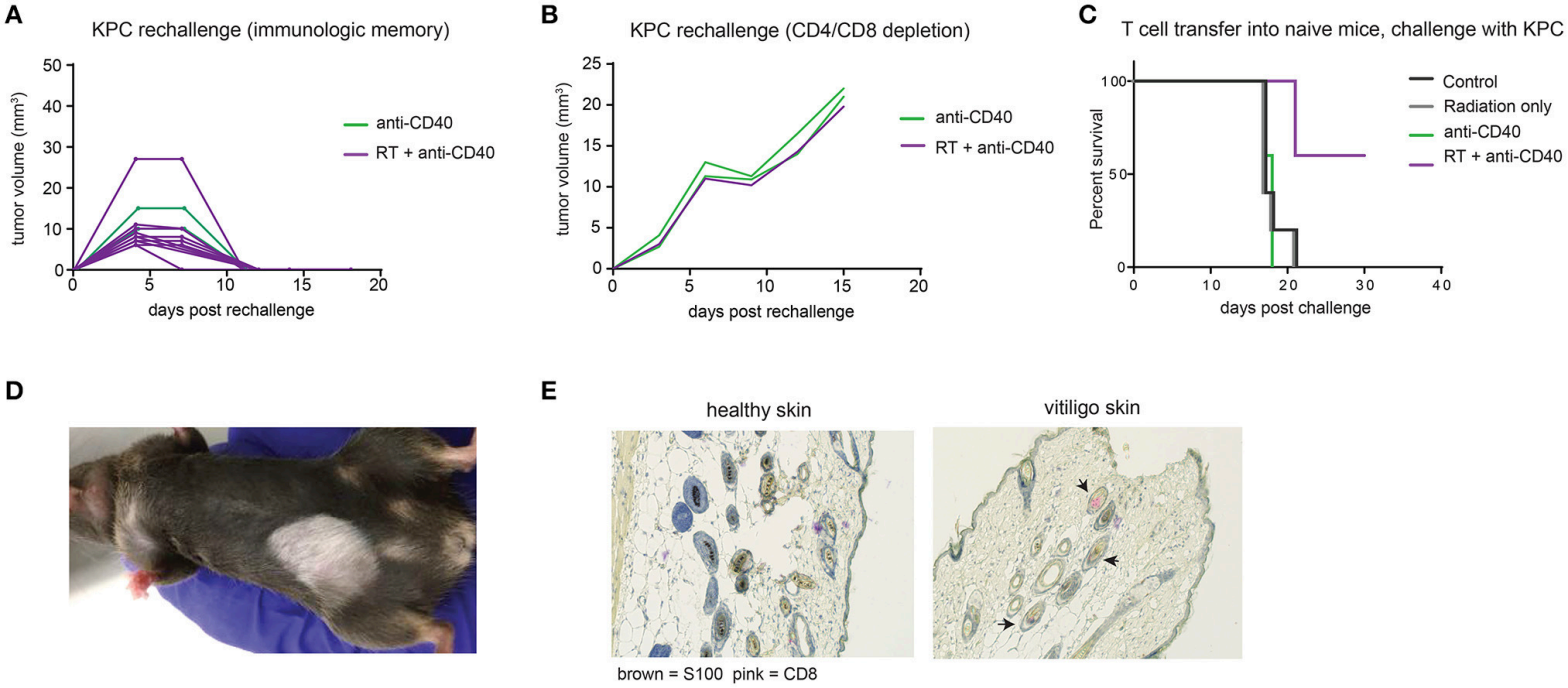
Combination RT and anti-CD40 induces immunologic memory and autoimmune vitiligo. **(A)** Mice from Figure 4 that were cured of their tumors by anti-CD40 or RT+anti-CD40 were rechallenged with 400,000 KPC cells in the absence of further treatment. **(B)** Mice from Figure 4 that were cured of their tumors were treated with depleting antibodies to CD4 and CD8 (100 μg every 3–4 days) and inoculated with 500,000 KPC cells. **(C)** Mice were treated as in Figure 4 with RT, anti-CD40 or RT+anti-CD40. Twelve days post treatment, spleens and lymph nodes were harvested, and total T cells isolated by magnetic bead selection. T cells from the indicated groups of donor mice were transferred into naïve recipient C57BL/6 mice that were then challenged with 200,000 KPC cells subcutaneously. Tumor growth was monitored until all mice were euthanized or tumor-free. **(D)** Representative picture of vitiligo development in combination treated mice that had been rechallenged with KPC tumors. **(E)** Histology of skin from an untreated mouse or a mouse with vitiligo shown in **(C)**. Immunohistochemistry stains for CD8 (pink) and the melanocyte marker S100 (brown). Arrowheads indicate CD8 staining in the hair follicles. Representative of 3 mice per group.

Intratumoral anti-CD40 was more effective in the KPC as compared to the Panc02 model, and long term survivors were observed in both anti-CD40 single agent and in the combination treated groups. To determine whether tumor regression was associated with induction of immunologic memory, surviving mice were rechallenged with a higher dose of KPC cells (4 × 10^5^). All mice rejected rechallenge in the absence of further treatment, indicative of immunologic memory (Figure 5A). To determine whether T cells were required for the immunologic memory observed, cured mice that survived rechallenge were depleted of CD4 and CD8 T cells and again rechallenged with a two-fold dose of KPC cells. Although all of these mice had demonstrable immunologic memory, T cell depletion allowed for outgrowth of KPC tumors in all cases (Figure 5B). Memory T cells generated in combination treated mice are superior to mice treated with single agent alone. We collected CD4 and CD8 T cells from mice 12 days after therapy and transferred these into naïve recipient hosts. We then challenged the new hosts with KPC tumors and found that only mice receiving T cells from combination treated donors were protected from tumor growth (Figure 5C). Thus we confirm that memory T cells capable of preventing tumor recurrence are generated with combination of SBRT and intratumoral anti-CD40.

Mice that had been treated with combination SBRT anti-CD40 also developed vitiligo at the site of rechallenge (Figure 5D). Vitiligo was not observed in mice that received radiation only and were monitored for 8 weeks following SBRT, suggesting that radiation-induced tissue damage was not responsible for depigmentation. Immunohistochemistry of affected skin revealed CD8 T cells residing in the hair follicles (Figure 5E and Supplemental Figure 2). Vitiligo responses have been reported previously in both mice and humans with melanoma treated with checkpoint blockade (42, 43), usually explained by T cells primed against self antigens shared between melanoma and melanocytes (44). In this case, we postulate that SBRT may be inducing death of surrounding normal tissues, and antigens from dying melanocytes may be acquired by dendritic cells. Antigen presentation is enhanced by anti-CD40, suggesting a means for development of autoreactive CD8 T cells, and ensuing destruction of healthy melanocytes by memory CD8 T cells recalled to the site of tumor rechallenge. Encouragingly, these autoreactive responses were restricted to melanocytes, as the skin epithelial cells and other normal tissues of the mouse were unaffected.

## Discussion

Radiation therapy is a promising adjunct to immunotherapy as it is widely used clinically and generates a source of immunogenic cell death. However, radiation treatment alone rarely generates productive CD8 T cell responses capable of clearing distant lesions. Case reports of abscopal effects induced in a few patients receiving checkpoint blockade prompted much excitement among clinicians (15, 18), although attempts to use SBRT to rescue patients who had failed ipilimumab (anti-CTLA-4) were less successful than might be hoped (16, 45). The sequence of radiation and immunotherapy, the SBRT dose and fractionation schedule, and which particular immunotherapy agent(s) are used likely make an enormous difference in the clinical outcome (46, 47). Indeed, we showed that multiple fractions of SBRT distributed over a week long period were far less effective in our Panc02 model in combination with anti-CD40 than a single SBRT dose. Other groups similarly reported a heavy reliance on timing and dose fractionation in mice, and clinical trials designed specifically with one or a few high doses of SBRT in combination with immunotherapy are now underway (48).

Currently approved checkpoint blockade therapies sustain productive T cell responses and can prevent or reverse T cell exhaustion. While certainly an important component of combination immunotherapy, checkpoint blockade does little to enhance dendritic cell activation, and T cell priming. To this end, local administration of adjuvants is most effective, and efforts to study combination of adjuvants with radiation have met with some success across a range of tumor types. Notably, STING agonists, TGFβ blockade, anti-CD40, checkpoint blockade, and TLR 7/8 ligands have been reported to synergize with radiation therapy in mice (12, 19, 35, 36, 49–51). We caution that the tumor microenvironments are different across different tumor types, and that agents used in one setting may not be amenable in another. TGFβ blockade, for example, although strikingly effective in combination with radiation in breast cancer (36, 41), had negligible effect in our KPC pancreatic tumor model, and in fact, reversed the efficacy of anti-CD40. We did observe improved local control of the treated tumors with anti-CD40, SBRT, and anti-TGFβ, with all mice fully clearing their tumors. Blockade of TGFβ signaling in pancreatic stellate cells promotes radiosensitivity (52), potentially rendering tumor cells better able to be cleared by CD40-activated local macrophages. TGFb signaling also promotes fibroblast deposition of extracellular matrix, and interrupting this pathway is likely to be more effective in combination with locally delivered therapies (53, 54). However, these striking local effects did not translate to improved systemic immunity, since adding anti-TGFβ to combination SBRT and anti-CD40 resulted in progressive outgrowth of non-treated tumors. We selected anti-CD40 as a rational choice for combination with SBRT in the setting of pancreatic cancer due to previous activity of this agent in mouse models and human pancreatic cancer patients (21–23) and the potential for augmentation of T cell priming in combination with radiation (18, 25, 51). Although previous studies administered anti-CD40 systemically (18, 25, 51), we found that local injection into the irradiated tumor site required five-fold less antibody, and was still effective at generating T cell-mediated immunity.

While overall less toxic than conventional cancer therapies, immunotherapy is not without risk (55). The development of autoimmune vitiligo in mice treated with radiation and anti-CD40 underscores the fact that augmentation of T cell priming may induce priming of both autoreactive and tumor-reactive T cells. In some cases, these two groups may overlap; tumors often overexpress tissue-restricted self antigens that may be recognized by T cells. In general, central tolerance results in deletion of overtly self-reactive T cells during thymic development, but weakly self-reactive T cells, or T cells recognizing antigens not displayed in the thymus may escape into the periphery. Despite their relatively low affinity, these T cells may be useful components of the anti-tumor immune response (56), and priming self-reactive T cells may be the major mechanism by which radiation and anti-CD40 synergize. T cell priming may be too effective, as it is unlikely in this case that KPC pancreatic tumors and healthy melanocytes share common tumor rejection antigens. Indeed vitiligo has now been observed outside of melanoma, in patients treated with radiation or checkpoint blockade for other malignancies (55, 57, 58). Limiting the field of radiation and the damage to healthy tissues may be critical to restricting immune-related toxicities.

Local delivery of adjuvants is key for combination with radiation therapy. Adjuvants must be present at the site of cell death for activation of tumor-antigen loaded dendritic cells (1, 7). Although intratumoral injection has thus far been attempted in melanoma, lymphoma, head and neck cancer and other tumors with skin-accessible lesions, technologies for local delivery to other sites are progressing. Interventional radiologists currently can access nearly any site for biopsy or placement of fiducial markers. Local adjuvants that can be administered with, or incorporated into, fiducial markers may be a practical approach for clinical delivery in combination with radiation therapy to generate *in situ* cancer vaccines.

## Materials and methods

### Cell culturing

Panc02 was obtained from the National Cancer Institute (59). KPC cells derived from a *LSL-Kras;p53*+*/floxed,Pdx-cre* mouse were a gift from Dr. Anirban Maitra (MD Anderson). Cells were cultured at 37°C in a humidified incubator with 5% CO_2_. RPMI media was supplemented with 10% FBS, 2 mmol/L L-glutamine, 1% penicillin/streptomycin, 1% MEM non-essential amino acids, 1 mmol/L sodium pyruvate, and 0.1 mmol/L β-mercaptoethanol. Cells used for *in vivo* experiments had been passaged for less than 2 months, were negative for known mouse pathogens, and were implanted at >95% viability.

### Mouse pancreatic subcutaneous tumor model

Female 6–8 week old C57BL/6J mice purchased from Jackson labs and used for KPC experiments. Panc02 experiments were replicated in both C57BL/6J (Jackson Labs) and C57BL/6NTac mice from Taconic. Syngeneic Panc02 or KPC cells were inoculated subcutaneously into both flanks of wild-type C57BL/6 mice at 2 × 10^5^ or 1.5 × 10^5^ cells, respectively. When tumors reached palpable size (week 2–3), mice were randomized and treatments were administered. Mice were observed at least twice per week and tumor measurements were performed using precision calipers at least once per week. In some experiments, CT scans were periodically performed to corroborate manual measurements. Mice were euthanized when either tumor exceeded 1 cm in diameter, or when tumors ulcerated. For mice that were cured of their initial tumors and rechallenged with KPC cells, 5 × 10^5^ cells were inoculated. Animals were maintained and experiments were conducted at the DFCI Animal Resources Facility in accordance with IACUC guidelines. Animals were treated according to protocols approved by the Dana-Farber Cancer Institute IACUC.

### Radiation therapy (RT) and CT image analysis

A Small Animal Radiation Research Platform (SARRP) was used to administer RT at 220 kVp and 13 mA using either a 10 × 10 or 5 × 5 mm collimator and a 0.15 mm copper filter. Mice were anesthetized with isoflurane and image-guided RT was used to specifically irradiate tumors on the right flank. Panc02 tumors receiving a single dose of radiation were given 5 Gy whereas KPC tumors were given 10 Gy. For cohorts receiving fractionated radiation, a total of 30 Gy was administered over the course of three (10 Gy × 3) or six (5 Gy × 6) consecutive days. Whole-body CT images were manually segmented using Preclinical Imalytics Software (developed at ExMI, Aachen, Germany, along with Philips Research, Aachen, Germany) (60), allowing three-dimensional measurement of tumor volume.

### Antibodies

Monoclonal anti-CD40 (clone FGK, BioXcell) was injected intratumorally into the treated tumors of relevant mice. Anti-CD40 or PBS was administered either as a single 20 μg dose or as two 10 μg doses spaced 3 days apart as indicated in the figure legends. Mice receiving both RT + anti-CD40 were treated with anti-CD40 within 3 h after radiation was administered.

### Histopathology

Tumors from both flanks, as well as lung tissue in applicable cases, were extracted and fixed in 10% formalin. Sections were stained with hematoxylin and eosin (H&E), and images were obtained using an Eclipse E1000M microscope (Nikon). For CD8 immunohistochemistry, paraffin-embedded tumor tissue was sliced into 5 μm-thick sections with a microtome, air-dried, fixed with acetone, and stained by the DFCI Rodent Histopathology Core. Immunostaining was performed using anti-CD8 (Abcam) according to the manufacturer's protocol. Multi-color images were obtained using a Zeiss fluorescent microscope.

### Flow cytometry

Tumors were extracted from mice, digested in RPMI supplemented with type II collagenase (Sigma) and soybean trypsin inhibitor (Life Technologies), and dispersed into a single-cell suspension by filtering with a 40 micron cell strainer. Cell preparations were stained and analyzed using a Sony spectral cytofluorimeter (SP6800). Flow cytometry antibodies used in this study were purchased from BioLegend (anti-CD45-BV711 [clone 30-F11], anti-CD11c-APC [N418], anti-CD11b-FITC [M1/70], anti-Gr-1-PE-Cy7 [RB6-8C5], anti-I-A/I-E-BV510 [M5/114.15.2], anti-CD4-BV421 [GK1.5], anti-CD103-PE [2E7], anti-B220-BV605 [RA3-6B2], anti-Ly6C-BV570 [HK1.4], anti-CD8-PacificBlue [53-6.7]).

### Statistical analysis

Groups were compared using a two-tailed Student's *t*-test. All reported tests were two-tailed and were considered significant at *p* < 0.05. Survival assays were plotted using Graphpad Prism and were analyzed using Log-rank (Mantel-Cox) and Gehan-Breslow Wilcoxon tests. Error bars are SD unless otherwise noted.

## Author contributions

SY-K, PB, and SG designed and performed experiments and analyzed data. MM, SK, and GZC performed experiments. RK contributed to experimental design. SD and WN supervised the project, analyzed data, and wrote the manuscript with input from all of the authors.

### Conflict of interest statement

The authors declare that the research was conducted in the absence of any commercial or financial relationships that could be construed as a potential conflict of interest.

## Supplementary material

Supplemental Figure 1 Pancreatic tumors are resistant to radiation. **(A)** Mice bearing palpable subcutaneous KPC tumor were treated with the indicated doses of SBRT. Tumors were harvested 14 days later. **(B)** Mice bearing palpable subcutaneous Panc02 tumors were treated with the indicated doses of SBRT. Tumor growth was monitored over time.

Supplemental Figure 2 Histology of control and vitiligo skin. Skin from 8 week old untreated C57BL/6 mice, contralateral skin from a mouse with vitiligo, and white vitiligo skin were paraffin-embedded, sectioned and stained with antibodies to CD8 (pink) and the melanocyte marker S100 (brown). Two representative images are shown in Figure 5.
