# Supplementary material for: Radiation and Local Anti-CD40 Generate an Effective in situ Vaccine in Preclinical Models of Pancreatic Cancer
Source: Front Immunol. 2018 Sep 7;9:2030. doi: 10.3389/fimmu.2018.02030 (PMC6137176; doi:10.3389/fimmu.2018.02030)
Supplement: Supplemental Figure 1 — Pancreatic tumors are resistant to radiation. (A) Mice bearing palpable subcutaneous KPC tumor were treated with the indicated doses of SBRT. Tumors were harvested 14 days later. (B) Mice bearing palpable subcutaneous Panc02 tumors were treated with the indicated doses of SBRT. Tumor growth was monitored over time. [file Data_Sheet_1.PDF]

A

KPC tumors treated Day 12, harvested Day 24

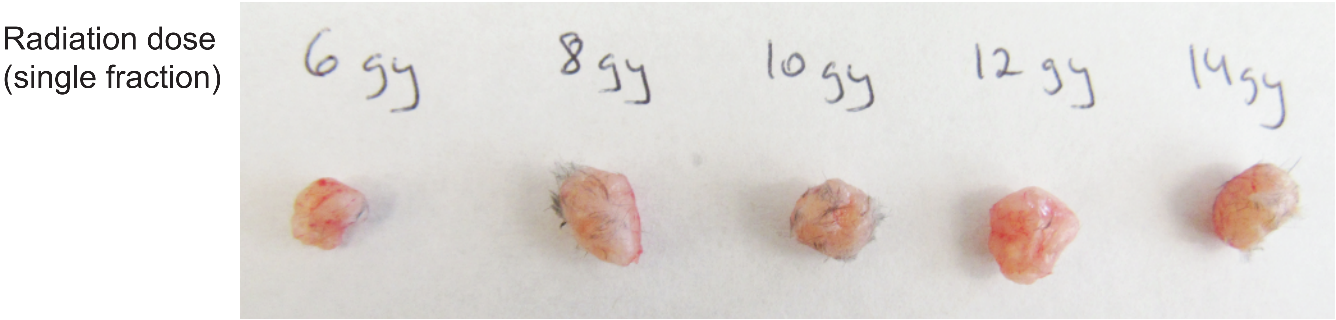

B

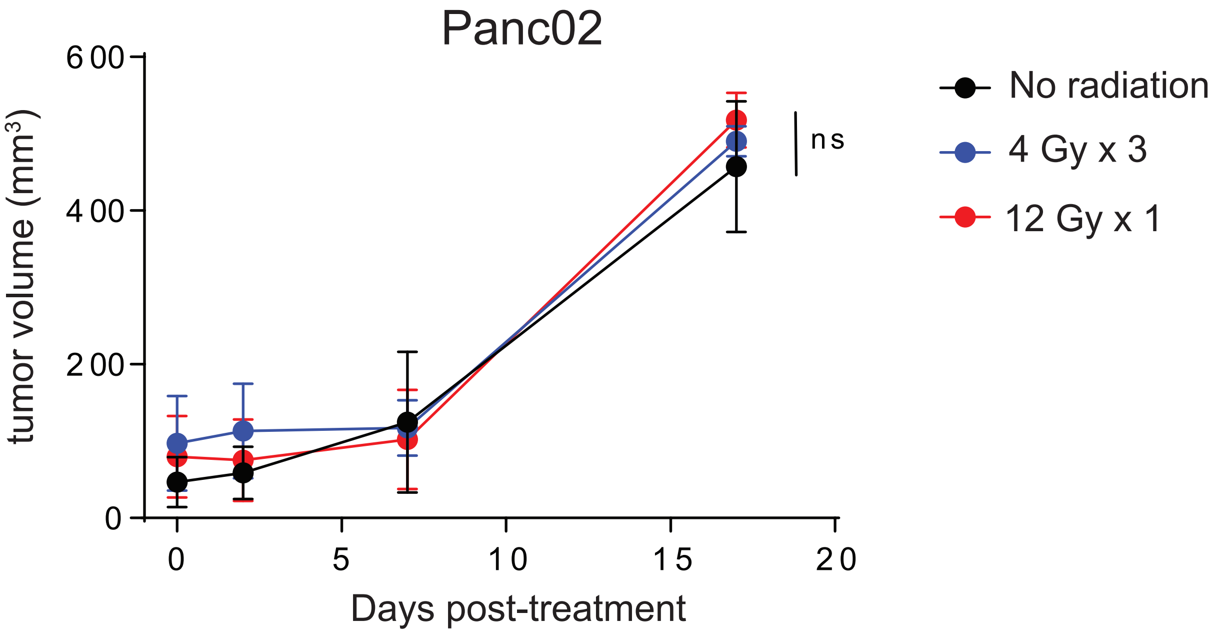

Supplemental Figure 1

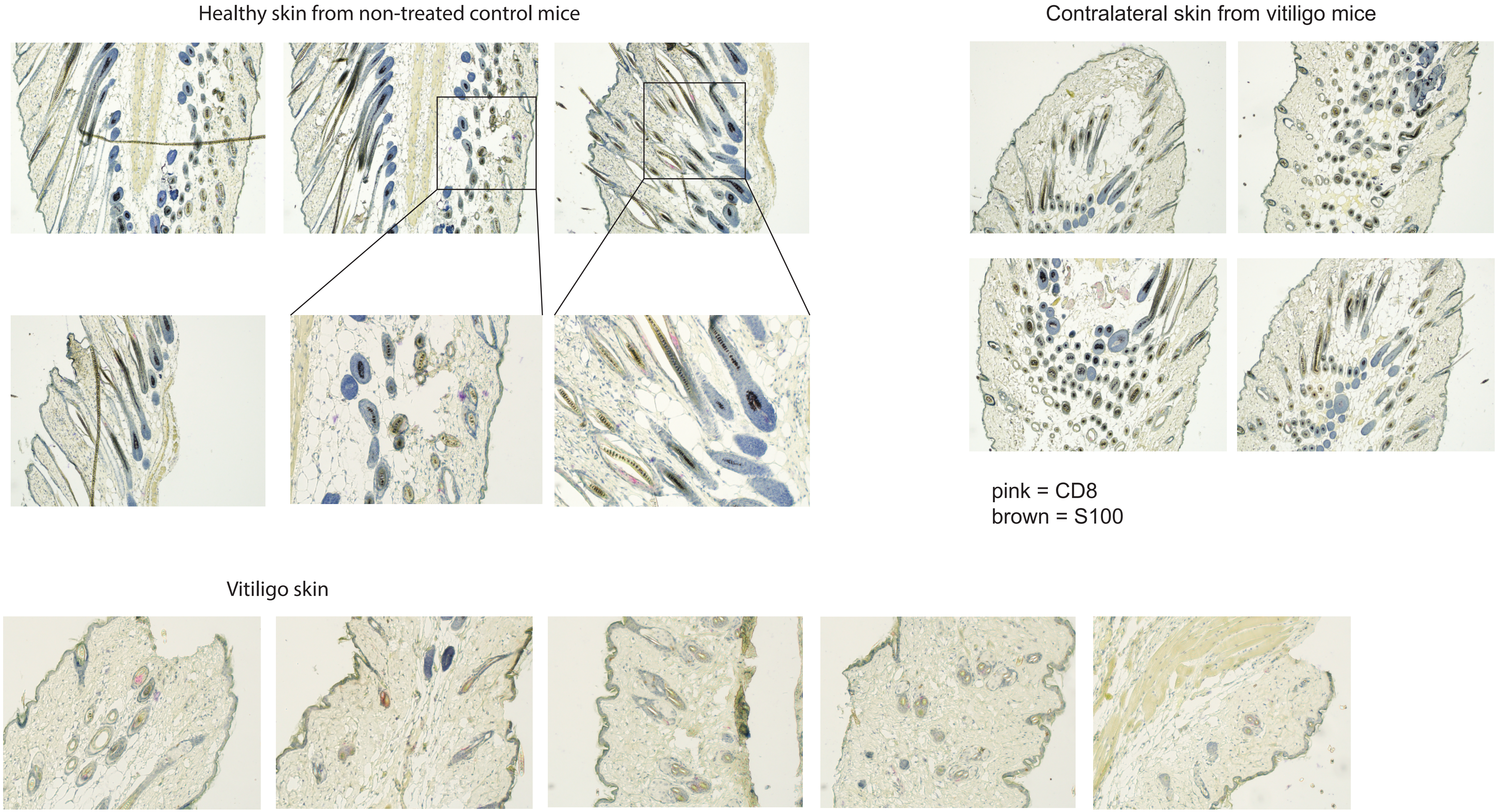

Supplemental Figure 2
